# Informal institutions and corporate carbon emissions: Evidence from China’s listed companies

**DOI:** 10.1371/journal.pone.0341892

**Published:** 2026-02-03

**Authors:** Shanshuai Hou

**Affiliations:** School of Economics, Southwestern University of Finance and Economics, Chengdu, China; University of Naples Federico II: Universita degli Studi di Napoli Federico II, ITALY

## Abstract

This research explores the connection between religious culture and carbon emissions through the lens of informal institutions, offering valuable insights into the shift to a green economy in China. The research sample comprises listed enterprises from 2010–2020 to investigate the influence of indigenous religious culture, represented by Buddhism and Taoism, on the corporate carbon footprint. The results reveal that religious culture has a notable inhibitory effect on corporate carbon outputs. Through a suite of robustness checks, the findings remain in line with the benchmark results. Heterogeneity analysis reveals that the effect of religious culture on corporate carbon emissions is more noticeable in economically thriving areas, state-owned companies, polluting-intensive companies, capital-intensive companies, and high-tech companies. Mechanistic tests suggest that religious culture mainly reduces corporate carbon emissions through three pathways: promoting corporate social responsibility, alleviating corporate financing constraints, and increasing corporate green innovation. Research has shown that both formal institutions and foreign cultural impacts weaken the beneficial impact of indigenous religious culture on corporate carbon reduction but do not eliminate its effect. This study provides practical guidelines and theoretical references for enterprises and policy-makers in addressing climate change and achieving sustainable development.

## Introduction

Since China's reform and opening-up, rapid economic growth has been heavily reliant on fossil energy consumption. This dependence, however, has led to persistently high carbon dioxide emissions, seriously challenging the nation's dual carbon goals (carbon peaking and carbon neutrality) [1] and threatening its sustainable development trajectory [2,3]. As the world's largest energy consumer and carbon emitter [4], China faces intense international pressure to curb its emissions [5,6]. Enterprises, as primary economic actors, account for approximately 70% of energy use and over 50% of greenhouse gas emissions [7,8], making them crucial to achieving national climate targets. In response, the Chinese government has implemented numerous environmental regulations aimed at reducing corporate carbon emissions. However, the effectiveness of these formal institutions is often limited by implementation challenges and design flaws [9,10], resulting in significant variation in firms' emission behaviors [11,12]. These facts indicate that formal institutions alone cannot fully explain corporate carbon performance, pointing to the potential importance of informal institutional factors.

Informal institutions, particularly religious culture with deep social roots and normative influence, may complement formal regulations [13]. In many contexts, religion has demonstrated policy-shaping power—Weber famously called it "a powerful driving force of history" [14]—and recent work links religious engagement to climate governance and behavioral adaptation worldwide [15–20]. Religion can shape environmental attitudes and corporate decisions through value formation, ethical education, and social norms. Although recent studies have begun to examine how informal institutions affect corporate environmental behavior [21–24], evidence on the role of religious culture in corporate pollution control—especially carbon emissions—remains scarce. Existing research on religion and the environment is dominated by Western settings and delivers mixed conclusions: while some scholars document a "greening" trend in religious engagement [25–33], others find neutral or even negative environmental effects associated with certain doctrines and communities [34–39]. The indigenous Chinese religions of Buddhism and Daoism, which emphasize harmony between humanity and nature [40–44], have received far less attention. Prior work suggests they may influence corporate environmental responsibility practices in China [45–47], but we still lack systematic evidence on how local religious culture shapes firms' carbon emission decisions. Clarifying this relationship is essential for understanding whether indigenous religions are becoming an effective informal force for carbon mitigation in China.

To address this gap, we examine whether—and through which channels—indigenous Chinese religious culture curbs corporate carbon emissions. We assemble a firm-level panel of A-share listed companies from 2010 to 2020 and, drawing on a geographic proximity framework, construct a fine-grained measure of local religious culture by calculating the density of Buddhist temples and Taoist shrines surrounding each firm's office location. We merge this measure with manually collected corporate carbon emission data and a rich set of financial and regional controls to estimate the impact of religious culture on firm-level carbon outcomes. Our research shows that religious culture in firms’ office locations negatively affects corporate carbon emissions, and this result remains robust after a series of endogeneity tests. The carbon-reducing effect is more pronounced among firms located in economically developed regions, state-owned enterprises, pollution-intensive industries, capital-intensive firms, and high-tech firms. Mechanism tests reveal that religious culture operates through three pathways: enhancing corporate social responsibility, alleviating financing constraints, and fostering green innovation. Further analysis indicates that formal institutions and exogenous cultural shocks can weaken, but not eliminate, the beneficial influence of indigenous religious culture on corporate carbon reduction.

This study makes three key contributions to the literature. First, it is among the earliest to systematically examine the influence of religious culture—a foundational yet underexplored informal institution—on corporate carbon emission disparities. While prior research has identified numerous determinants of firms' carbon emissions—spanning internal facets (e.g., firm profitability [48], cash holdings [49], export orientation [50,51], R&D and innovation [52–55], executive traits [56–62], governance and strategy [63–65], and CSR [66,67]) and external facets (e.g., distance [68], stakeholders [69–73], green finance [74–76], policy and institutional environment [77–82] and political factors [83–85])—far less attention has been paid to the role of informal cultural systems [10,45–47]. By conceptualizing religion as a cultural driver of corporate environmental behavior, this study helps fill this theoretical gap and broadens the scope of carbon reduction research.

Second, prior studies primarily connect religious culture with corporate financial decision-making [86–89], risk preferences and managerial behavior [90–93], corporate governance [94], corporate social responsibility [95–97], innovation [98–100], and accounting or auditing practices [101–104], yet they seldom test whether religion shapes firms’ environmental externalities. We extend this literature by identifying the causal impact of local religious culture on corporate pollution behavior, using firm-level carbon emissions as the outcome and an instrumented fine-grained measure of the density of Buddhist temples and Taoist shrines for identification. This perspective fills a clear research gap and supplies both theoretical and empirical support for understanding how informal institutions can promote corporate carbon reduction.

Third, this research identifies and validates the mechanisms through which religious culture reduces corporate carbon emissions. Our findings demonstrate that religious culture strengthens corporate social responsibility, mitigates financing constraints, and stimulates green innovation—collectively forming a transmission channel through which cultural values are translated into concrete emission reductions. By empirically establishing these pathways, this study provides a coherent theoretical framework for understanding how informal institutions shape corporate environmental conduct, while also offering actionable insights for policymakers and firms designing effective low-carbon strategies.

The organization of our study is outlined as follows: The second section elaborates on the theoretical framework and formulates the research hypotheses. The third section focuses on describing the data and methodologies employed. The fourth section examines the empirical findings and their interpretations, whereas the final section concludes with a summary of the main insights and implications.

## Theoretical framework and hypothesis development

### Environmental protection concepts in indigenous Chinese religious culture

Buddhism and Daoism are deeply woven into China's cultural fabric and are often described as "green religions" because their cosmologies emphasize the interdependence of all life [44,105]. Buddhist ideas of dependent origination and nonself view all beings as mutually connected and equally deserving of respect, encouraging stewardship of the natural environment [43,45,106,107]. Daoism anchors this ethic in the maxim "Dao follows nature," stressing harmony with natural laws and reverence for mountains, forests, and land as living systems [44,108]. These traditions supply cultural narratives that legitimize modern environmental protection and help embed low-carbon norms in communities and organizations.

### Theoretical analysis of religion and corporate carbon emissions

In the following section, we aim to explore how religion influences corporate carbon emission behaviors and propose corresponding hypotheses on the basis of this exploration.

Religious teachings impose moral expectations that can temper environmentally harmful behavior by managers and employees [45,47]. In regions steeped in Buddhist or Daoist values, firms face community pressure to respect nature, making emission reduction a tangible expression of moral integrity. Religious communities also cultivate shared social norms that influence adherents and non-adherents alike [95,101,109,110]. Such norms can sanction polluting behavior and reward responsible practices, pushing firms toward lower emissions. Religious groups further provide moral oversight through collective action, demanding transparency and accountability on environmental performance [111,112]. Finally, religious culture elevates corporate social responsibility (CSR) standards, nudging companies to disclose environmental data and adopt proactive emission-control measures [10,45–47]. Taken together, these ethical, normative, and community mechanisms imply that stronger religious culture lowers corporate carbon intensity. Therefore, we propose Hypothesis 1:

Religious culture is negatively associated with corporate carbon emissions.

### Theoretical logic of enterprise carbon reduction

Religious culture embeds a value system that primes firms to assume social responsibility, thereby enabling carbon abatement [45,47,101]. This value orientation then operates through concrete CSR channels: to safeguard legitimacy and reputation, firms disclose environmental information, intensify pollution control, and steer financial resources toward green innovation in response to stakeholder expectations [66,67,113–118]. Strengthened CSR, in turn, secures credibility and policy or capital access while deploying green technologies and efficiency upgrades that directly lower energy use and emissions. Under China's "dual-carbon" targets, the social norms and moral surveillance nurtured by religious culture magnify this sequence, consolidating a pathway of "religious culture → strengthened CSR → green transition → carbon reduction". Building upon the aforementioned analysis, we formulate Hypothesis 2:

Religious culture reduces corporate carbon emissions by strengthening corporate social responsibility.

Religious culture mitigates corporate financing constraints and thereby curbs carbon emissions. Financial access is critical for funding low-carbon technology and green investment [119,120], and religious norms of integrity and social responsibility help firms secure funding by cultivating moral reputations and supportive financing environments [86–88,117]. China's green finance policies underscore this linkage: capital increasingly favors firms with strong environmental credentials, steering them toward green transitions [74–76,121,122]. Firms steeped in religious culture tend to pursue energy efficiency, emission cuts, and clean energy, which elevates their credibility, lowers agency risk, and increases alignment with green finance criteria [43,88,123]. Initiatives such as the Green Credit Policy tighten credit for polluters, while improving financing efficiency and R&D investment among compliant firms, further strengthening green innovation and carbon-reduction capacity [122,124]. Building upon the aforementioned analysis, we formulate Hypothesis 3:

Religious culture reduces corporate carbon emissions by alleviating financing constraints.

Religious culture stimulates corporate green innovation, thereby shrinking carbon footprints. Green innovation—encompassing clean energy adoption, energy-efficiency upgrades, and low-pollution processes—is recognized as central to sustainable, low-carbon development yet remains underprovided because of its risk, capital intensity, and long horizons [52–55,125]. Religious norms of natural harmony and ecological stewardship embed moral obligations in corporate strategy, prompting managers to prioritize environmental choices and absorb the costs of low-carbon technologies and clean production [99]. These norms also deepen social trust, curb moral hazard, and tighten governance structures that nurture green R&D. The collaborative ethos of religious communities further encourages experimentation in high-risk, high-spillover domains, raising firms’ willingness to invest in green technological innovation [99,126]. Building upon the aforementioned analysis, we formulate Hypothesis 4:

Religious culture reduces corporate carbon emissions by increasing green technological innovation.

## Data and methods

### Data sources and sample extraction

This study selected all A-share listed companies from 2010 to 2020 as the research sample and applied the following screening criteria: (1) excluding financial companies; (2) excluding samples of companies whose trading status is ST, *ST, PT, or in the delisting consolidation period; (3) excluding samples of companies whose debt-to-assets ratio is either at least 1 or exactly 0; and (4) excluding samples with severe missing values. After applying these criteria, the final sample comprises an unbalanced panel of 15,569 firm-year observations from 2,392 listed companies.

The data for this study are sourced from the following: (1) Buddhist temple and Taoist shrine data from the CNRDS database; (2) corporate carbon emissions data, manually collected from listed companies' annual, social responsibility, and environmental reports; (3) firm-level control variables derive from the CSMAR database; and (4) city-level control variables come from the China Urban Statistical Yearbook.

### Model specification

This paper uses a multidimensional fixed effects model to test the research hypotheses and constructs the following model:


$${CEI}_{i,t}=\beta_{\text{0}}+\beta_{\text{1}}{Religion}_{i,\;t}+\beta_{\text{2}}{Control}_{i,t}+\beta_{\text{3}}{Control}_{c,t}+\mu_{i}+\delta_{industry}+\gamma_{t}+\varepsilon_{i,t}$$ (1)

where, i, c and t respectively represent enterprises, cities, and years, respectively, and *CEI*_*i,t*_ is the explained variable, representing the carbon emissions of enterprises, expressed by the intensity of carbon dioxide emissions. *Religion*_*i,t*_ is the key explanatory variable, indicating the atmosphere of religious culture. *Control*_*i,t*_ and *Control*_*c,t*_ represent the control variables at the enterprise level and at the city level, respectively. $\mu_{i}$ is the enterprise fixed effect, *δ*_*industry*_ is the industry fixed effect, $\gamma_{t}$ is the year fixed effect, and $\varepsilon_{i,t}$ is the random disturbance term.

### Variable description

#### Explained variable.

Carbon Intensity of Enterprise Emissions (CEI). We measure corporate carbon emissions using data disclosed in listed firms' annual reports, corporate social responsibility reports, sustainability reports, or environmental reports. For firms that do not directly report total emissions, we calculate direct and indirect carbon emissions based on their consumption of various fossil fuels, electricity, and heat, following the "Guidelines for Accounting and Reporting of Greenhouse Gas Emissions for Enterprises" issued by China's National Development and Reform Commission. We then aggregate these values to obtain total corporate carbon emissions. Consistent with prior research [70], our proxy for carbon emission intensity is the natural logarithm of carbon dioxide emissions per unit of revenue.

#### Core explanatory variable.

Religious Culture (Religion). Existing literature commonly measures regional religious intensity using the number of religious sites, the proportion of adherents in the population, or frequency of participation in religious activities. However, these approaches are less suitable for capturing Buddhist and Taoist influence in China. Unlike many Western religions, neither Buddhism nor Taoism maintains formal membership registries, making it difficult to accurately count followers. Moreover, religious attendance is generally less frequent among Chinese adherents, further limiting the applicability of participation-based metrics. To more accurately capture firms' exposure to local religious culture, we follow prior studies [10,96] and use the number of religious sites within a specific radius of a firm's headquarters. Specifically, our proxy for religious culture is the natural logarithm of the total number of Buddhist temples and Taoist shrines located within 150 km of a company's office location.

#### Control variables.

To mitigate the potential impact of omitted variables, we incorporate a set of control variables at both the firm and city levels, following established practices in the literature. Firm-level controls include: (1) Firm size (Size), measured as the natural logarithm of total assets; (2) Leverage ratio (Lev), defined as total liabilities divided by total assets; (3) Return on equity (ROE), represented by net profit divided by equity; (4) Board size (Board), measured as the total number of board members; (5) CEO–chair duality (Dual), an indicator that equals 1 if the CEO also serves as board chair, and 0 otherwise; (6) Tobin's Q; (7) Listing age (ListAge), calculated as the natural logarithm of (current year − listing year + 1); (8) Female executive ratio (Female), measured as the proportion of female executives; Bank–enterprise relationship (Bank), an indicator that equals 1 if the firm holds bank shares, and 0 otherwise; Revenue growth rate (Growth), defined as (current year revenue − prior year revenue)/ prior year revenue; (11) Environmental investment (EPI), measured as the natural logarithm of environmental protection investment; (12) R&D intensity (RD), represented by the ratio of R&D personnel to total employees; (13) Cash flow ratio (Cashflow), defined as net operating cash flow divided by total assets; (14) Environmental concern (EC), an indicator that equals 1 if the firm discloses environmental, safety, or quality issues, and 0 otherwise; Proportion of independent directors (Indep), measured as the ratio of independent directors to total board members; (16) Proportion of executives with environmental backgrounds (EEBR), calculated as the ratio of such executives to the total number of executives. City-level control variables include: (1) Urban GDP (GDP_city), measured as the natural logarithm of municipal GDP; (2) Urban population (POP_city), represented by the natural logarithm of the year-end residential population; (3) Urban industrial structure (IS_city), defined as the ratio of secondary industry value-added to tertiary industry value-added. Table 1 presents the descriptive statistics for the primary variables.

**Table 1:** Descriptive statistics.

Variable	Obs	Mean	SD	Min	Max
CEI	15569	5.778	.15	5.172	9.933
Religion	15569	6.268	1.144	1.099	8.449
Size	15569	22.235	1.323	17.806	28.636
Lev	15569	.409	.197	.008	.995
EC	15569	.266	.442	0	1
RD	15569	.064	.316	0	13.64
ROE	15569	.066	.146	−4.32	2.379
EPI	15569	19.615	1.192	16.25	25.794
TOP1	15569	34.715	14.553	2.866	89.093
EEBR	15569	.079	.169	0	1
Dual	15569	.283	.45	0	1
Bank	15569	.062	.241	0	1
Board	15569	2.129	.196	1.386	2.89
Indep	15569	37.533	5.648	18.18	80
TobinQ	15569	2.03	1.629	.684	92.25
Female	15569	18.087	10.992	0	68.42
Growth	15569	.211	1.659	−.913	96.024
ListAge	15569	2.076	.785	0	3.401
Cashflow	15569	.051	.067	−.528	.664
IS_city	15569	.902	.45	.187	7.669
POP_city	15569	6.369	.678	.942	8.136
GDP_city	15569	8.722	1.05	3.983	10.377

## Empirical results and analyses

### Main results

Table 2 presents the baseline regression results examining the relationship between religious culture and corporate carbon emission intensity. Column (1) includes religious culture along with firm, industry, and year fixed effects, while Column (2) additionally controls for firm-level and city-level characteristics. Across both specifications, religious culture exhibits a statistically significant negative association with carbon emission intensity (at the 1% level), thereby confirming Hypothesis 1. From an economic standpoint, a 1% increase in religious culture is associated with a 0.038% reduction in emission intensity. This finding is consistent with the results of Li et al. [10] and aligns with the theoretical view that religious ethics and social norms collectively shape corporate environmental behavior [109,110]. Specifically, religious teachings emphasize harmony with nature, fostering a moral commitment to environmental protection. Simultaneously, the social norms fostered by religious communities create an external supervision mechanism that deters unethical corporate practices [45–47]. Taken together, these channels encourage firms to adopt greener practices and ultimately reduce their carbon emissions.

**Table 2:** The influence of religious culture on corporate carbon emissions.

	(1)	(2)	(3)	(4)
Variables	CEI			
	All		Buddhism	Taoism
Religion	−0.0339***	−0.0380***	−0.0393***	−0.0299***
	(-3.7965)	(-6.3588)	(-6.1998)	(-8.5802)
Size		−0.0366***	−0.0366***	−0.0369***
		(-5.5967)	(-5.6177)	(-5.5443)
Lev		−0.0077	−0.0069	−0.0072
		(-0.6338)	(-0.6362)	(-0.6103)
Bank		−0.0227***	−0.0227***	−0.0228***
		(-4.4529)	(-4.5033)	(-4.3035)
ROE		−0.0348***	−0.0347***	−0.0348***
		(-5.5013)	(-5.5630)	(-5.3241)
TOP1		−0.0008***	−0.0008***	−0.0008***
		(-3.4275)	(-3.4001)	(-3.4495)
EEBR		0.0142**	0.0136**	0.0135**
		(2.8479)	(2.7969)	(2.8087)
EC		0.0141***	0.0144***	0.0140***
		(8.3754)	(8.4330)	(8.2405)
Board		0.0096	0.0093	0.0101
		(0.8640)	(0.8489)	(0.9155)
TobinQ		0.0017***	0.0017***	0.0016***
		(3.8663)	(3.8828)	(3.8029)
Indep		0.0006***	0.0006***	0.0006***
		(3.3060)	(3.3137)	(3.2955)
Dual		0.0037**	0.0036**	0.0036**
		(2.7792)	(2.7525)	(2.7534)
Cashflow		−0.0109	−0.0109	−0.0109
		(-0.4789)	(-0.4777)	(-0.4829)
Growth		−0.0054*	−0.0054*	−0.0053*
		(-1.9135)	(-1.9143)	(-1.9117)
ListAge		0.0142***	0.0141***	0.0142***
		(5.6265)	(5.6056)	(5.7344)
RD		0.0121***	0.0121***	0.0122***
		(3.9370)	(3.9325)	(3.9609)
EPI		−0.0153***	−0.0152***	−0.0149***
		(-4.3868)	(-4.3969)	(-4.3545)
Female		−0.0000	−0.0000	−0.0000
		(-0.0050)	(-0.0043)	(-0.0722)
IS_city		−0.0063	−0.0067	−0.0068
		(-0.5913)	(-0.5914)	(-0.6282)
POP_city		0.0036	0.0038	0.0048
		(0.8325)	(0.9046)	(1.3999)
GDP_city		−0.0334**	−0.0353**	−0.0304**
		(-2.7608)	(-2.8777)	(-2.6038)
Constant	5.9905***	7.3586***	7.3671***	7.2146***
	(107.1066)	(35.3536)	(34.5953)	(38.0263)
Firm_FE	√	√	√	√
Industry_FE	√	√	√	√
Year_FE	√	√	√	√
Observations	15569	15569	15569	15569
R-squared	0.163	0.181	0.181	0.181

Note: *** denotes p < 0.01, ** indicates p < 0.05, and * represents p < 0.1. Values in parentheses represent the t-statistics, based on industry-clustered standard errors.

We next examine the distinct impact of Buddhist and Taoist cultures on corporate carbon emissions. As reported in Columns (3) and (4), both traditions significantly reduce corporate carbon emission intensity, though the influence of Buddhist culture is statistically stronger. This finding aligns with Su [46] who also reports Buddhism's more pronounced effect on corporate social responsibility. The stronger association for Buddhism may be attributed to doctrinal differences: Buddhist teachings on "dependent origination" and "nonself" directly emphasize the interdependence of all beings, explicitly advocating for resource conservation and environmental protection. Taoism, though likewise pro-environmental, focuses on following natural laws ("ziran"), and often exerts influence through indirect channels. Overall, these results not only confirm the carbon-reduction effect of religious culture but also highlight its role in promoting corporate environmental practices through moral restraint, societal norms, and external oversight. Collectively, this provides a theoretical basis for understanding how religious institutions can motivate firms to adopt social responsibility and reduce pollution.

Regression analysis of the control variables yields several insights. Larger firm size significantly mitigates carbon emission intensity, suggesting that resource and technological advantages facilitate emission reduction. Similarly, higher EPI significantly lowers emissions, highlighting the efficacy of dedicated environmental expenditures. In contrast, listing age is positively associated with emission intensity, implying that mature firms may be less responsive to environmental policies. The positive coefficient on EEBR suggests that this characteristic has not consistently translated into emission reduction outcomes, potentially due to organizational or market constraints. Notably, R&D intensity increases emission intensity, implying that firms' innovation activities may be concentrated in non-environmental fields or involve energy-intensive processes in the short term. At the regional level, GDP is negatively associated with emission intensity, consistent with stricter environmental enforcement in more developed areas. City population size, however, shows no significant effect, indicating that demographic scale bears no direct relationship to corporate emission behavior. Regarding financial performance, ROE reduces emission intensity, implying that profitable firms are better positioned to undertake environmental initiatives. Conversely, the positive effect of Tobin's Q suggests that firms with high market valuation may prioritize short-term financial performance over long-term environmental investments. Collectively, these findings underscore the multifaceted nature of corporate emission behavior and highlight the need for differentiated policy approaches.

### Robustness test results

#### Radius for changing religious sites.

To assess robustness, we alternately employ radii of 60 km to 300 km to measure religious culture. The 300 km upper bound is justified by the concept of "daily accessibility" in transport geography, where a two- to three-hour travel circle defines a critical threshold for routine cross-city interaction [127]—a scope that corresponds directly to the "two-hour interconnectivity within city clusters" target outlined in China's national transport strategy. This spatial scale is consistent with established practices in the literature on regional culture, wherein a 300 km radius has been effectively employed to capture the influence of religious atmosphere [128]. These frequent population movements serve as a vehicle for transmitting cultural norms, including religious beliefs and practices. Thus, the 300 km radius (approximately 1.5–2 hours by high-speed rail) is designed to capture religious cultural effects within a plausibly integrated region, as defined by both theoretical and policy frameworks. The regression results for these alternative radii are presented in Table 3. Results indicate that our core findings are robust across all alternative radius choices.

**Table 3:** Robustness test: the impact of the radius of different religious sites on regression results.

	(1)	(2)	(3)	(4)	(5)	(6)
Variables	CEI					
	R = 60 km	R = 80 km	R = 100 km	R = 200 km	R = 250 km	R = 300 km
Religion	−0.0252***	−0.0317***	−0.0356***	−0.0406***	−0.0431***	−0.0464***
	(-6.4163)	(-7.8343)	(-8.8551)	(-5.8577)	(-6.7659)	(-7.4778)
Constant	7.2216***	7.2734***	7.3104***	7.3921***	7.4313***	7.4611***
	(37.4315)	(37.5682)	(37.6826)	(35.1427)	(35.9298)	(35.4801)
CVs	√	√	√	√	√	√
Firm_FE	√	√	√	√	√	√
Industry_FE	√	√	√	√	√	√
Year_FE	√	√	√	√	√	√
Observations	15569	15569	15569	15569	15569	15569
R-squared	0.180	0.181	0.181	0.181	0.181	0.181

Note: *** denotes p < 0.01, ** indicates p < 0.05, and * represents p < 0.1. Values in parentheses represent the t-statistics, based on industry-clustered standard errors. This table summarizes all the variables mentioned in Table 1. Because of space constraints, the coefficients for the control variables (CVs) have been omitted.

#### Replacing the dependent variable.

To further assess the broader environmental implications of religious culture, we replace carbon dioxide emissions in our baseline model with three alternative measures: nitrogen oxide emissions in industrial wastewater, sulfur dioxide emissions, and particulate matter emissions. As reported in Table 4, religious culture exhibits a robust negative association with all three alternative pollution measures, significant at the 1% or 5% level. These consistent results indicate that the inhibitory effect of religious culture extends beyond carbon emissions to encompass a wider range of corporate pollutants.

**Table 4:** Robustness analysis: replacing the dependent variable.

	(1)	(2)	(3)
Variables	lnSo2_intensity	lnNox_intensity	lnSmoke_intensity
Religion	−0.0684**	−0.0911***	−0.0777***
	(−2.9588)	(−3.9714)	(−3.0791)
Constant	20.2843***	20.7162***	21.0740***
	(28.3214)	(29.8580)	(28.1750)
CVs	√	√	√
Firm_FE	√	√	√
Industry_FE	√	√	√
Year_FE	√	√	√
Observations	15370	15370	15370
R-squared	0.973	0.974	0.974

Note: *** denotes p < 0.01, ** indicates p < 0.05, and * represents p < 0.1. Values in parentheses represent the t-statistics, based on industry-clustered standard errors. This table summarizes all the variables mentioned in Table 1. Because of space constraints, the coefficients for the control variables (CVs) have been omitted.

#### Reducing sample size.

To address potential endogeneity between religious culture and corporate carbon emissions, we implement three sample restrictions: (1) excluding firms headquartered in minority autonomous regions, where distinctive religious beliefs and complex cultural landscapes could confound our results; (2) dropping firms that relocated during the sample period, as such moves could introduce unrelated confounding influences; and (3) retaining only firms listed before the sample period began to ensure a consistent and stable sample. As reported in Columns (1) to (3) of Table 5, the estimated coefficients on religious culture remain statistically significant and stable in magnitude across these alternative samples. This consistency confirms that our baseline findings are robust to these sample refinements.

**Table 5:** Robustness analysis: reducing sample size and IV.

	(1)	(2)	(3)	(4)
Variables	CEI			
	Exclude Minority Regions	Remove migrated enterprises	Balance check 2010–2020	2SLS
Religion	−0.0299***	−0.0431***	−0.0132**	−0.3985**
	(-4.5062)	(-5.5546)	(-2.7740)	(-1.9985)
CVs	√	√	√	√
Firm_FE	√	√	√	√
Industry_FE	√	√	√	√
Year_FE	√	√	√	√
Observations	15035	15191	7295	15442
First-stage regression results				
Variables				Religion
IV				0.0045*** (4.5779)
Underidentification test				24.765
Weak instruments test				20.927

Note: *** denotes p < 0.01, ** indicates p < 0.05, and * represents p < 0.1. Values in parentheses represent the t-statistics, based on industry-clustered standard errors. This table summarizes all the variables mentioned in Table 1. Because of space constraints, the coefficients for the control variables (CVs) have been omitted.

#### Constructing instrumental variables.

This paper identifies the ruggedness of urban terrain as an instrumental variable for religious culture. The variable satisfies the standard conditions of relevance and exogeneity. On the relevance side, Buddhist temples and Taoist shrines are mostly located in mountainous areas of China [129]. The Chinese saying "Famous mountains are often occupied by monks" is relevant because site selection for Taoist shrines and Buddhist temples has long favored scenic, spiritually significant hills and valleys far from urban dust to enhance their mystical aura. Consequently, areas with greater terrain ruggedness tend to host more temples and shrines, increasing firms' exposure to religious culture. On the exogeneity side, terrain ruggedness is an exogenous, time-invariant geographic feature of cities that should not directly affect corporate carbon emissions, making it a suitable instrument. Because terrain ruggedness reflects inherent city characteristics that do not change over time, we introduce exogenous time variation by interacting it with year dummies. Therefore, following Duflo et al. [130], we use this interaction as an instrumental variable to identify the effect of religious culture on corporate carbon footprints.

The two-stage least squares (2SLS) estimation results are reported in Column (4) of Table 5. In the first stage, the instrumental variable displays a strong positive correlation with religious culture (p < 0.01). The F-statistic of 20.927 in the weak instrument test exceeds the conventional threshold of 10, allowing us to reject the null hypothesis of a weak instrument. These results suggest that our instrumental variable is both valid and strong. The second-stage analysis thus confirms that religious culture continues to exhibit a statistically significant suppressive effect on corporate carbon emissions.

### Heterogeneity analyses

Having documented evidence that local religious culture reduces corporate carbon emissions, we now examine how this effect varies across key firm and regional characteristics.

#### Heterogeneity analyses of different regional enterprises.

Given China's substantial regional variation in religious and cultural ambiance, we examine how its influence on corporate carbon emissions differs across the eastern, central, western, and northeastern regions. Columns (1) to (4) of Table 6 report the corresponding regression results. These reveal a significant inhibitory effect of religious culture on corporate carbon emissions only in the eastern region, with no statistically meaningful impact observed in the central, western, or northeastern parts of the country. This regional disparity may be explained by the eastern region’s comparative advantages in economic development, social conditions, and policy implementation. Within this more developed institutional setting, religious culture can more effectively serve as a guiding and motivating force, prompting firms to place greater emphasis on environmental responsibility and adopt more proactive carbon reduction measures. By contrast, the central, western, and northeastern regions generally exhibit lower levels of economic development, weaker policy enforcement, and less mature social infrastructure. Although religious culture is present in these areas, its influence on corporate emission behavior seems to be constrained by these broader structural limitations, thereby attenuating its observable effect.

**Table 6:** Heterogeneity analysis of different regional and pollutant-intensive enterprises.

	(1)	(2)	(3)	(4)	(5)	(6)
Variables	CEI					
	East	Central	West	NorthEast	PIEs	Non-PIES
Religion	−0.0488***	−0.0155	−0.0176*	0.0018	−0.0428***	−0.0285***
	(−4.8708)	(−1.7937)	(−4.0039)	(0.0902)	(−14.3126)	(−3.4447)
Constant	7.1906***	7.2740***	5.9784***	6.5032***	6.9362***	7.1721***
	(43.5525)	(17.9834)	(35.5516)	(32.8785)	(153.3495)	(24.9570)
CVs	√	√	√	√	√	√
Firm_FE	√	√	√	√	√	√
Industry_FE	√	√	√	√	√	√
Year_FE	√	√	√	√	√	√
Observations	10,566	2,361	1,817	632	6,385	9,143
R-squared	0.175	0.252	0.486	0.149	0.198	0.187

Note: *** denotes p < 0.01, ** indicates p < 0.05, and * represents p < 0.1. Values in parentheses represent the t-statistics, based on industry-clustered standard errors. This table summarizes all the variables mentioned in Table 1. Because of space constraints, the coefficients for the control variables (CVs) have been omitted.

#### Heterogeneity analyses of different polluting intensive enterprises.

The influence of religious culture on corporate carbon emissions also varies by pollution intensity. Based on the "Environmental Audit Classification Management Catalogue for Listed Companies" (Ministry of Environmental Protection, 2008), which identifies 14 polluting industries, we classify firms into pollution-intensive enterprises (PIEs) and non-PIEs. Columns (5) and (6) of Table 6 present the results by pollution intensity. The regression results show that religious culture significantly inhibits corporate carbon emissions in both groups, although the effect is more pronounced in PIEs. This stronger effect in PIEs can be attributed to their higher emission levels, which subject them to stricter regulatory scrutiny and public pressure. Confronted with stringent environmental requirements, PIEs are more likely to respond to the environmental ethics in religious culture by adopting stricter and more effective emission reduction measures. Although non-PIEs face lower pollution levels and regulatory pressure, religious culture still exerts a significant mitigating influence on their carbon emissions. Nevertheless, given their inherently lower emission base, the relative effect size is smaller than that observed in PIEs.

#### Heterogeneity analyses of different types of enterprise ownership.

Given their distinct governance structures, management models, and institutional backgrounds, state-owned enterprises (SOEs) and non-SOEs may adopt different strategies for addressing climate change and reducing carbon emissions. Columns (1) and (2) of Table 7 report the regression results by ownership type. These show a significant negative relationship between religious culture and corporate carbon emissions among SOEs, whereas no such association is observed for non-SOEs. This suggests that the inhibitory effect of religious culture on corporate carbon emissions is unique to SOEs. A plausible explanation is that, unlike non-SOEs, SOEs are strongly influenced by national policies and are expected to serve as tools for strictly implementing government agendas [131]. In the context of the "double carbon" goals, SOEs are mandated to prioritize social responsibility and public interests, positioning them as leaders in executing environmental policies [132]. Accordingly, SOEs are more likely to incorporate the environmental values inherent in religious culture into their management practices, thereby adopting more proactive carbon reduction measures. In contrast, non-SOEs may focus more on economic benefits and market competition, granting them greater flexibility in responding to climate challenges and mitigating greenhouse gas emissions; As a result, religious culture exerts a less pronounced influence on the carbon footprint of non-SOEs.

**Table 7:** Analysis of Heterogeneity in Enterprises with Varying Ownership and Factor Intensity.

	(1)	(2)	(3)	(4)	(5)
Variables	CEI				
	SOEs	Non-SOEs	LIEs	CIEs	TIEs
Religion	−0.0873***	0.0029	−0.0378	−0.0543***	−0.0026***
	(−10.4411)	(0.2911)	(−1.0491)	(−32.7105)	(−15.9087)
Constant	7.6713***	7.1192***	6.4213***	6.9739***	6.4167***
	(53.4743)	(23.4654)	(12.0444)	(88.1211)	(130.6252)
CVs	√	√	√	√	√
Firm_FE	√	√	√	√	√
Industry_FE	√	√	√	√	√
Year_FE	√	√	√	√	√
Observations	5,124	10,097	3,056	4,186	8,269
R-squared	0.203	0.193	0.195	0.206	0.203

Note: *** denotes p < 0.01, ** indicates p < 0.05, and * represents p < 0.1. Values in parentheses represent the t-statistics, based on industry-clustered standard errors. This table summarizes all the variables mentioned in Table 1. Because of space constraints, the coefficients for the control variables (CVs) have been omitted.

#### Heterogeneity analyses of different factor-intensive enterprises.

The impact of religious culture on corporate carbon emissions varies significantly across firms with different factor intensities. Following the classification of the sample into labor-intensive, capital-intensive, and high-tech companies, Columns (3) to (5) in Table 7 present the corresponding regression results. These reveal a pronounced suppressive effect of religious culture on corporate carbon emissions in capital-intensive and high-tech companies, but not in labor-intensive companies. This heterogeneity can be attributed to the stronger resource base and technological capabilities of capital-intensive and high-tech companies, which enable them to better respond to environmental norms and pressures. First, capital-intensive companies typically possess greater financial capacity to allocate to eco-friendly technologies and process upgrades. Religious culture can motivate these companies to prioritize social and environmental responsibilities, thereby encouraging proactive investment in carbon-abatement technologies. Second, high-tech companies rely on innovation and advanced technology to maintain competitiveness, making them more receptive to adopting new environmental technologies and low-carbon methods. The ethical norms advocated by religious culture can further stimulate their enthusiasm for green innovation, leading to more substantial carbon reduction outcomes. By contrast, labor-intensive firms tend to focus more on labor costs and short-term economic benefits often at the expense of environmental and social responsibility. As a result, the carbon-reduction effect of religious culture is statistically insignificant in labor-intensive firms.

### Mechanism analysis and in depth exploration

#### Mechanism analysis.

Having established that religious culture reduces corporate carbon emissions, we now examine the mechanisms underlying this effect. Based on the theoretical framework in Section 2, we focus on three potential channels: enhanced corporate social responsibility (CSR), mitigated financing constraints, and the promotion of green innovation.

The results in Column (1) of Table 8 demonstrate that religious culture significantly strengthens corporate social responsibility (CSR). The moral tenets of religious traditions—such as compassion and integrity—motivate firms to proactively address their social duties [46,96], which in turn trigger greater environmental protection efforts and lower carbon emissions [66,67]. This finding validates Hypothesis 2, confirming that religious culture curbs emissions by enhancing CSR.

**Table 8:** Mechanism test.

	(1)	(2)	(3)	(4)
Variables	CSR	FC	Greenpatent1	Greenpatent2
Religion	0.8236**	−0.0020***	0.5777**	0.3196**
	(2.8690)	(−3.6529)	(2.3318)	(2.6765)
Constant	−42.5586***	0.0767***	−12.7436*	−6.7394*
	(−12.6164)	(3.6088)	(−1.7805)	(−2.1439)
CVs	√	√	√	√
Firm_FE	√	√	√	√
Industry_FE	√	√	√	√
Year_FE	√	√	√	√
Observations	15,562	12,748	15,569	15,569
R-squared	0.605	0.960	0.822	0.798

Note: *** denotes p < 0.01, ** indicates p < 0.05, and * represents p < 0.1. Values in parentheses represent the t-statistics, based on industry-clustered standard errors. This table summarizes all the variables mentioned in Table 1. Because of space constraints, the coefficients for the control variables (CVs) have been omitted.

Column (2) of Table 8 indicates that religious culture significantly alleviates financing constraints. By bolstering a firm's social reputation, religious adherence facilitates access to green financing [86–88]. Such financial support enables more efficient capital allocation and fosters low-carbon innovation and green investment, thereby reducing emissions [117,122,124], in line with Hypothesis 3.

Columns (3) and (4) further show that religious culture stimulates green innovation. This outcome stems from several factors outlined in our theoretical framework: a heightened sense of CSR directs managerial attention toward eco-friendly decisions [99]; increased social trust and reduced moral hazard improve corporate governance, creating an environment conducive to innovation [94]; and a cooperative ethic encourages firms to pursue high-risk green technological exploration [99,125,126]. Together, these forces drive green innovation and support Hypothesis 4.

#### In depth exploration.

As a cornerstone of Chinese traditional ethics, local religious culture exerts a non-negligible influence on microeconomic actors. This section investigates the heterogeneous effects of religious culture on corporate carbon emissions through the lenses of institutional substitution and cultural shocks.

Can religious culture effectively substitute for formal institutions? New institutional economics suggests that informal institutions often play an irreplaceable role where formal institutions are weak [133]. To test this proposition, we introduce interaction terms between religious culture and various dimensions of formal institutions. A positive coefficient on the interaction term would indicate a substitution effect, whereas a negative one would suggest complementarity.

We measure formal institutions in several ways. First, corporate environmental investment captures the stringency of environmental regulations (ER) [132]. Column (1) of Table 9 reports a significantly positive interaction term between religious culture and this measure, indicating substitution. Second, we adopt the marketization index (MI), legal system environment index (LSEI), and nonstate-owned economy development index (non-SOEDI) from the "Marketization Index for China's Provinces: Neri Report 2021" to represent the market development environment, rule of law environment, and property rights safeguard environment, respectively [16]. As shown in Columns (2) to (4) of Table 9, the interaction terms with religious culture are consistently positive and significant. This pattern confirms that the carbon-reduction effect of religious culture is stronger in regions with less developed markets, weaker legal systems, and poorer protection of property rights—supporting the substitution hypothesis and aligning with prior research [16,59,124].

**Table 9:** Deeper exploration: the dynamics with formal institutions.

	(1)	(2)	(3)	(4)
Variables	CEI			
	ER	MI	Non-SOEDI	LSEI
Religion	−0.1318**	−0.0555***	−0.0580***	−0.0448***
	(-2.5814)	(-5.3211)	(-7.7735)	(-6.3689)
Formal Institution	−0.0448**	−0.0154*	−0.0131**	−0.0062*
	(-2.9206)	(-1.9042)	(-2.5382)	(-1.8018)
Religion×Formal Institution	0.0048*	0.0022*	0.0021***	0.0007*
	(1.9275)	(1.8868)	(3.0411)	(1.7742)
Constant	7.9185***	7.4644***	7.4782***	7.3588***
	(17.6802)	(30.8914)	(30.5671)	(32.5152)
CVs	√	√	√	√
Firm_FE	√	√	√	√
Industry_FE	√	√	√	√
Year_FE	√	√	√	√
Observations	15,569	15,569	15,569	15,569
R-squared	0.181	0.181	0.181	0.181

Note: *** denotes p < 0.01, ** indicates p < 0.05, and * represents p < 0.1. Values in parentheses represent the t-statistics, based on industry-clustered standard errors. This table summarizes all the variables mentioned in Table 1. Because of space constraints, the coefficients for the control variables (CVs) have been omitted.

To examine whether foreign cultural influences weaken the effect of local religious culture, we consider China's modern history of port openings, which facilitated the influx of Western ideologies and religions. Specifically, we focus on Christianity and Catholicism as key vectors of Western religious culture. We measure the presence of Western religions using the city-level number of Christian and Catholic churches (data from CNRDS) and employ a coastal open city indicator to capture broader exposure to foreign culture. We then construct interaction terms between indigenous religious culture and these two proxies for foreign cultural impact.

As reported in Table 10, the coefficients on both indigenous religious culture and Western cultural exposure are strongly negative, whereas the interaction terms are positive and significant at the 1% level. These results show that foreign cultural influences significantly attenuate the positive role of local religious traditions in promoting corporate carbon reduction.

**Table 10:** Further analysis: interaction effects with foreign cultures.

Variables	(1)	(2)
	CEI	
	Open	Christianity
Religion	−0.0438***	−0.0505***
	(-6.4742)	(-8.1684)
Foreign Culture	−0.2330***	−0.0009***
	(-3.1327)	(-9.2594)
Religion×Foreign Culture	0.0316***	0.0001***
	(4.3899)	(19.2455)
Constant	7.3708***	7.4703***
	(40.2563)	(36.1639)
CVs	√	√
Firm_FE	√	√
Industry_FE	√	√
Year_FE	√	√
Observations	15,569	15,569
R-squared	0.181	0.182

Note: *** denotes p < 0.01, ** indicates p < 0.05, and * represents p < 0.1. Values in parentheses represent the t-statistics, based on industry-clustered standard errors. This table summarizes all the variables mentioned in Table 1. Because of space constraints, the coefficients for the control variables (CVs) have been omitted.

## Conclusions

This study shows that indigenous Buddhist and Taoist traditions function as informal institutions that restrain Chinese firms' carbon emissions by fostering corporate social responsibility, alleviating financing constraints, and catalyzing green innovation. The effect is strongest among state-owned enterprises, firms in economically advanced regions, and pollution-intensive or high-tech industries, highlighting the conditional power of cultural forces. Furthermore, while formal institutions and foreign cultural influences can attenuate this positive impact, they do not negate it, underscoring the resilience of indigenous cultural forces.

Theoretically, this study integrates religious culture into the new institutional economics framework for environmental governance, offering a cultural account of interfirm emission disparities and explicating the process by which informal norms translate into managerial behaviors.

For policymakers, our findings argue for the strategic incorporation of local religious resources to complement formal regulations, particularly where the latter are weak. Augmenting this approach with targeted CSR guidance and green R&D incentives will accelerate the low-carbon transition.

The primary limitation of this work is its focus on Buddhist and Taoist contexts within Chinese listed firms; future research should explore other religious traditions, more diverse samples, and longer time frames to establish the persistence and generalizability of these cultural effects.

## Supporting information

S1 File Data. (XLSX)
